# Evaluation of Medical-Grade Polycaprolactone for 3D Printing: Mechanical, Chemical, and Biodegradation Characteristics

**DOI:** 10.3390/polym17202730

**Published:** 2025-10-11

**Authors:** Eun Chae Kim, Jae-Seok Kim, Yun Jin Yu, Sang-Gi Yu, Dong Yeop Lee, Dong-Mok Lee, So-Jung Gwak, Kyoung Duck Seo, Seung-Jae Lee

**Affiliations:** 1 Department of Mechanical Engineering, Wonkwang University, 460 Iksandae-ro, Iksan 54538, Republic of Korea; kimeunchae1008@naver.com (E.C.K.); jaeseokkim311@gmail.com (J.-S.K.); 98box@naver.com (S.-G.Y.); 2 Division of Mechanical Engineering, Wonkwang University, 460 Iksandae-ro, Iksan 54538, Republic of Korea; biology0509@gmail.com (Y.J.Y.); kdlehdduq@naver.com (D.Y.L.); 3 Korea Institute on Industrial Technology, Yeongcheon 38822, Republic of Korea; cowboyle@kitech.re.kr; 4 Department of Chemical Engineering, Wonkwang University, 460 Iksandae-ro, Iksan 54538, Republic of Korea; plus38317@wku.ac.kr; 5 MECHABIO Group, Wonkwang University, 460 Iksandae-ro, Iksan 54538, Republic of Korea; 6 Advanced Bio-Convergence Research Center, Wonkwang University, 460 Iksandae-ro, Iksan 54538, Republic of Korea

**Keywords:** polycaprolactone (PCL), medical-grade, 3D printing, tissue engineering

## Abstract

Polycaprolactone (PCL) is one of the most widely used polymers in tissue engineering owing to its excellent biocompatibility, biodegradability, and processability. Nevertheless, most previous studies have primarily employed research-grade PCL, thereby limiting its clinical translation. In this study, four types of medical-grade PCL (RESOMER^®^ C203, C209, C212, and C217) were systematically evaluated for their applicability in three-dimensional (3D) printing, with respect to printability, mechanical characteristics, chemical stability, and biodegradation behavior. Among these, C209 and C212 exhibited superior printability and mechanical strength. FT-IR analysis showed that the chemical structure of PCL remained unchanged after both 3D printing and E-beam sterilization, while compressive testing demonstrated no significant differences in mechanical characteristics. In vitro degradation assessment revealed a time-dependent decrease in molecular weight. For kinetic analysis, both C209 and C212 were fitted using pseudo-first-order and pseudo-second-order models, which yielded comparable coefficients of determination (*R*^2^), suggesting that degradation may be governed by multiple factors rather than a single kinetic pathway. Taken together, these findings indicate that medical-grade PCL, particularly C209 and C212, is highly suitable for 3D printing. Furthermore, this study provides fundamental insights that may facilitate the clinical translation of PCL-based scaffolds for tissue engineering and biomedical implantation.

## 1. Introduction

Tissue engineering aims to regenerate or replace damaged tissues by integrating cells, scaffolds, and bioactive factors [1,2,3]. Within this field, three-dimensional (3D) printing has attracted increasing attention because it enables precise control over scaffold geometry, pore size, and mechanical properties [4,5,6]. This technology also allows the fabrication of patient-specific scaffolds with high reproducibility, thereby enhancing clinical applicability [7,8,9].

Polycaprolactone (PCL) is one of the most widely utilized polymers for scaffold fabrication owing to its favorable biocompatibility, biodegradability, and processability. Moreover, certain grades of PCL have been approved by the U.S. Food and Drug Administration (FDA) for medical device applications, underscoring its potential for clinical translation [1,10]. However, most previous studies have focused on research-grade PCL and demonstrated its efficacy in various in vitro [11,12,13] and in vivo models [14,15,16,17]. Research-grade PCL, however, does not meet the stringent manufacturing and quality standards required for clinical use under Good Manufacturing Practice (GMP).

In contrast, medical-grade PCL is produced under GMP conditions, ensuring high purity, safety, and traceability, and is therefore suitable for regulatory approval and clinical trials. Despite this, systematic investigations of the applicability of medical-grade PCL in 3D printing remain limited [18,19,20].

For clinical application, sterilization is another indispensable requirement to prevent microbial contamination. Among available methods, electron beam (E-beam) sterilization has been widely adopted for polymer-based medical devices because of its rapid processing, absence of toxic residual by-products, and ease of process control. However, the high-energy irradiation involved in E-beam sterilization has been reported to induce polymer chain scission, potentially reducing molecular weight and mechanical integrity [21,22]. This highlights the need for comprehensive evaluation of material properties following sterilization.

In this study, four grades of medical-grade PCL (RESOMER^®^ C203, C209, C212, and C217) were systematically investigated to assess their suitability for 3D printing applications. Following widely reported methods for 3D-printed polymer scaffolds, printability was evaluated by scanning electron microscopy (SEM) and dimensional metrics (e.g., porosity, pore area, and pore size) [23,24,25].

Because sterilization can induce chain scission and thereby alter mechanical and chemical properties, mechanical performance was characterized by tensile and compressive testing on a universal testing machine (UTM) [26,27,28], and chemical changes were monitored by Fourier-transform infrared spectroscopy (FT-IR) [29,30]. In addition, gel permeation chromatography (GPC) was used to track time-dependent changes in molecular weight in phosphate-buffered saline (PBS, 37 °C), thereby assessing biodegradation behavior [31,32].

By establishing comprehensive baseline data on medical-grade PCL, this study aims to provide fundamental insights that may facilitate regulatory approval and promote the clinical translation of PCL-based scaffolds for tissue engineering and biomedical implantation.

## 2. Materials and Methods

### 2.1. Materials

#### 2.1.1. Medical-Grade Polycaprolactone (PCL)

All medical-grade PCLs were purchased from Evonik (Essen, Germany) in pellet form, and their specifications are summarized in Table 1. Phosphate-buffered saline (PBS, pH 7.4) used for the degradation studies was obtained from ThermoFisher Scientific (St. Louis, MO, USA).

#### 2.1.2. Preparation of Specimens

**Figure 1:**
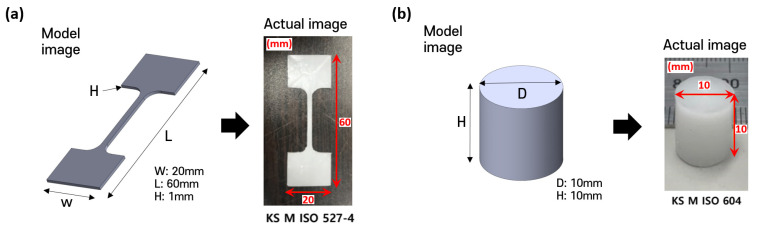
Preparation of specimens: (**a**) Tensile specimen (**b**) Compressive specimen.

To evaluate the mechanical properties of each material, standardized tensile and compressive specimens were fabricated. Tensile specimens were prepared using a metal mold (20 × 60 × 1 mm^3^; width × length × height) in accordance with KS M ISO 527-4 (Figure 1a) [33], while compressive specimens were fabricated using a cylindrical metal mold (10 mm in diameter and 10 mm in height) based on KS M ISO 604 (Figure 1b) [34]. PCL pellets were loaded into a 10 mL stainless steel barrel (SS10; U-Jin Tech., Siheung-si, Republic of Korea) and melted at 90 °C for more than 30 min. The molten PCL was slowly injected using a lab-made multi-head 3D bioprinting system into the molds through a 500 µm nozzle (PRN0.5; U-Jin Tech., Siheung-si, Republic of Korea) to minimize bubble entrapment. The filled molds were then placed on a hot plate at 90 °C for 2 h to remove residual bubbles, followed by cooling at room temperature for at least 12 h prior to demolding. For each material, three tensile and three compressive specimens (n = 3) were prepared to characterize the fundamental mechanical behavior of each material.

To evaluate the effects of sterilization on compressive strength, specimens were divided into four groups: (i) non-sterilized, (ii) pre-sterilized, (iii) post-sterilized, and (iv) both pre- and post-sterilized (Table 2). Pre-sterilization involved exposing PCL to E-beam irradiation prior to specimen fabrication, whereas post-sterilization was performed by E-beam irradiation of fabricated specimens. For each sterilization group, five specimens (n = 5) were prepared to ensure reliable comparison among groups. All specimens were prepared in the ISO-standard cylindrical form (10 mm in diameter and 10 mm in height), in accordance with KS M ISO 604.

### 2.2. Mechanical Testing

Mechanical testing was performed to evaluate the mechanical properties of each material. Tensile tests were conducted on a universal testing machine (UTM; Model E42, MTS, Berlin, Germany) equipped with a 5.0 kN load cell at a loading rate of 0.1 mm/s. Compressive tests were performed on the same instrument at a loading rate of 1 mm/min until 40% strain.

To assess the effect of sterilization on compressive strength, compression tests were carried out using a universal testing machine (ElectroPuls E10000, Instron, Norwood, MA, USA) equipped with a 10.0 kN load cell at a loading rate of 1 mm/min, up to 40% strain. Testing was conducted by the Korea Testing Laboratory (KTL, Gwacheon-si, Republic of Korea) in accordance with KS M ISO 604.

### 2.3. Extrusion and Printability Tests

#### 2.3.1. Extrusion Characteristics

The extrusion characteristics of the PCLs were evaluated using strand extrusion tests. Each sample, weighing 2 g, was loaded into a 10 mL stainless steel barrel (SS10; U-Jin Tech., Siheung-si, Republic of Korea) and melted at 90 °C for at least 30 min. The molten PCL was then extruded for 2 min under a pneumatic pressure of 550 kPa through a 500 µm nozzle (PRN0.2; U-Jin Tech., Siheung-si, Republic of Korea). The mass of the extruded strands was measured with an electronic balance (CB203; Mettler Toledo, Greifensee, Switzerland). Each condition was tested in quintuplicate.

#### 2.3.2. Printability

To assess printability, lattice-structured scaffolds were fabricated. Each PCL was loaded into a 10 mL stainless steel barrel and melted for more than 30 min at 90 °C for C209 and 120 °C for C212, depending on their viscosity. The molten PCL was extruded under a pneumatic pressure of 550 kPa through a 200 µm nozzle (PRN0.2; U-Jin Tech., Siheung-si, Republic of Korea) according to a predefined G-code to produce lattice scaffolds (5 × 5 × 3 mm^3^; width × length × height). The overall morphology was observed using an optical microscope (DMS1000; Leica, Wetzlar, Germany). The scaffolds were then sputter-coated with platinum for 60 s and examined using a field-emission scanning electron microscope (FE-SEM, S-4800; Hitachi, Tokyo, Japan) under an accelerating voltage of 5 kV, a working distance of 8.9 mm, and in secondary electron (SE) mode for analysis of microstructural and surface features. The obtained images were analyzed using the open-source software ImageJ 2.16.0 (National Institutes of Health, Bethesda, MD, USA).

### 2.4. Fourier-Transform Infrared (FT-IR) Analysis

Fourier-transform infrared (FT-IR) spectroscopy in attenuated total reflectance (ATR) mode was used to characterize the chemical structure of the specimens. The analysis was conducted by the Korea Testing & Research Institute (KTR, Gwacheon-si, Republic of Korea) in accordance with KS M 0024 [35]. Spectra were collected in the range of 4000–650 cm^−1^ with a resolution of 4 cm^−1^. FT-IR measurements were carried out using 3D-printed lattice scaffolds, as described in Section 2.3.2.

### 2.5. Degradation Assessment Methods

Degradation was assessed using scaffolds with the same dimensions as those described in Section 2.3.2. Each scaffold was immersed in PBS solution (pH 7.4) at 37 °C, and the PBS solution was refreshed weekly. Scaffolds were collected at predetermined time intervals and dried at room temperature. Molecular weight changes were analyzed by gel permeation chromatography (GPC, ACQUITY APC system; Waters, Beverly, MA, USA) using an APC XT 200 column (2.5 μm; flow rate 0.8 mL·min^−1^; injection volume 10 μL; run time 5 min) with refractive index (RI) detection at the Center for University-Wide Research Facilities (CURF), Jeonbuk National University.

All degradation data were analyzed using GraphPad Prism 10.4.1. For the pseudo-first-order model, the molecular weight at each time point was normalized to the initial molecular weight ($M_{w}$/$M_{w0}$) and fitted to an exponential decay function. For the pseudo-second-order model, reciprocal values ($\frac{1}{M_{w}(t)}-\frac{1}{M_{0}}$) were very small. Therefore, all data were multiplied by 10^5^ for clarity of visualization. Nonlinear regression was used to obtain the kinetic parameters (*k*, *k’*), and the quality of fit was assessed using the coefficient of determination ($R^{2}$).

### 2.6. Sterilization Methods

Sterilization was performed using electron beam (E-beam) irradiation at the Korea Institute of Industrial Technology (KITECH, Yeongcheon-si, Republic of Korea). The operating conditions were set to an electron beam energy of 5 MeV, a dose of 10 kGy, and a frequency of 25 Hz. Sterility assurance was validated by an external certified facility (QNAGMP, Anyang-si, Republic of Korea) in accordance with ISO 11737-2 [36], confirming that the scaffolds were successfully sterilized under these conditions.

### 2.7. Effect of Long-Term Thermal Exposure on Printability

The effect of extended melting on printability was investigated by processing C209 at two temperature conditions (90 °C and 140 °C) in a 10 mL stainless steel barrel. After approximately 30 min of melting, lattice scaffolds were fabricated in the form described in Section 2.3.2. This procedure was repeated every 12 h for up to 96 h. The weight ($m_{s}$) and volume ($V_{0}$) of each scaffold were measured using an electronic balance and a Vernier caliper, respectively. Porosity was calculated, assuming a PCL density ($\rho_{PCL}$ = 1.145 g/cm^3^) and determined when the printed constructs failed to form a cubic lattice structure.

(1) $$Porosity\;(\%)=\frac{{(V}_{0}-(m_{s}/\rho_{PCL}))}{V_{0}}\times 100(\%)$$

## 3. Results and Discussion

### 3.1. Tensile and Compressive Properties of PCLs

To compare the mechanical properties of the PCLs, tensile and compressive tests were conducted. Mechanical testing of C203 could not be performed due to its high brittleness. The specimens were damaged during molding and fractured during mounting on the testing machine. Therefore, the comparison focused on C209, C212, and C217.

**Figure 2:**
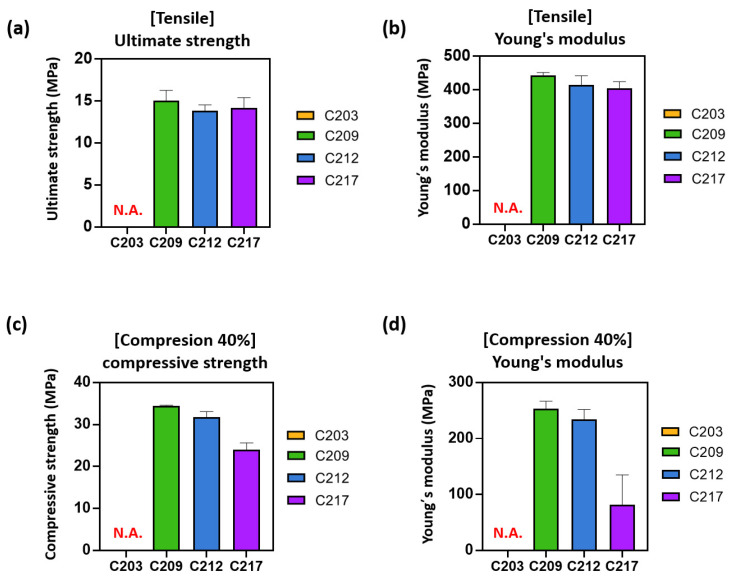
Tensile and compressive properties of polycaprolactone (PCL) (C203, C209, C212, C217): (**a**) Tensile strengths, (**b**) Tensile modulus, (**c**) Compressive strengths, and (**d**) Compressive modulus.

In the tensile test, the ultimate tensile strengths of C209, C212, and C217 were 15.0 ± 1.2, 13.8 ± 0.7, and 14.2 ± 1.2 MPa, respectively (Figure 2a). The corresponding Young’s moduli were 442.8 ± 8.6, 414.7 ± 27.0, and 405.2 ± 19.2 MPa (Figure 2b). In the compressive test, the compressive strengths were 34.4 ± 0.2, 31.8 ± 1.3, and 24.0 ± 1.7 MPa (Figure 2c), while the corresponding Young’s moduli were 258.7 ± 13.1, 234.5 ± 17.5, and 81.8 ± 53.4 MPa, respectively (Figure 2d).

The tensile results indicated that the ultimate strengths of the three PCLs were within the range of 13.8–15.0 MPa. Although C209 exhibited slightly higher values, the overall results were comparable. In contrast, distinct differences were observed in the compressive tests: C209 and C212 exhibited similar compressive strengths, whereas C217 showed a significantly lower value.

These findings suggest that the selection of medical-grade PCL for 3D printing may be influenced by the expected loading conditions. Therefore, careful evaluation of the mechanical characteristics of each material is essential for selecting the most suitable grade for biomedical applications and implantation environments.

### 3.2. Characteristics and Morphological Analysis of 3D-Printed PCLs

#### 3.2.1. Extrusion Properties of PCLs

**Figure 3:**
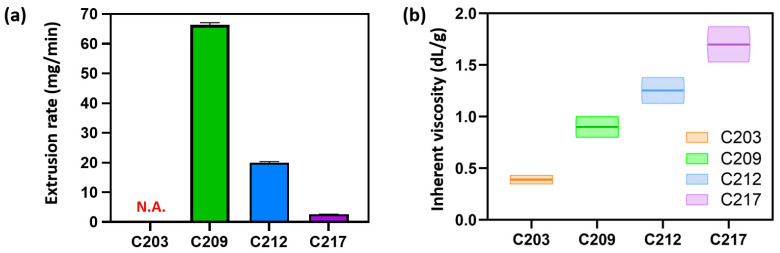
Comparison of extrusion properties: (**a**) Extrusion rate and (**b**) Inherent viscosity.

Comparison of extrusion rates revealed that C203 leaked from the nozzle even without pneumatic pressure. Upon applying pressure, the PCL was excessively extruded and rapidly depleted. As a result, stable strand formation was not achievable, and C203 was excluded from the comparison. The extrusion rates of C209 and C212 were 66.34 ± 0.7 and 20.0 ± 0.3 mg/min, respectively, whereas C217 exhibited a markedly lower extrusion rate of 2.6 ± 0.1 mg/min (Figure 3a).

These results revealed an inverse relationship with the intrinsic viscosity of each PCL material (Figure 3b), indicating that rheological properties strongly influence extrusion behavior and strand morphology even under identical printing conditions. Viscosity-driven differences in extrusion rates are likely critical factors of production time and the optimization of processing parameters.

#### 3.2.2. Printability Evaluation of PCL

**Figure 4:**
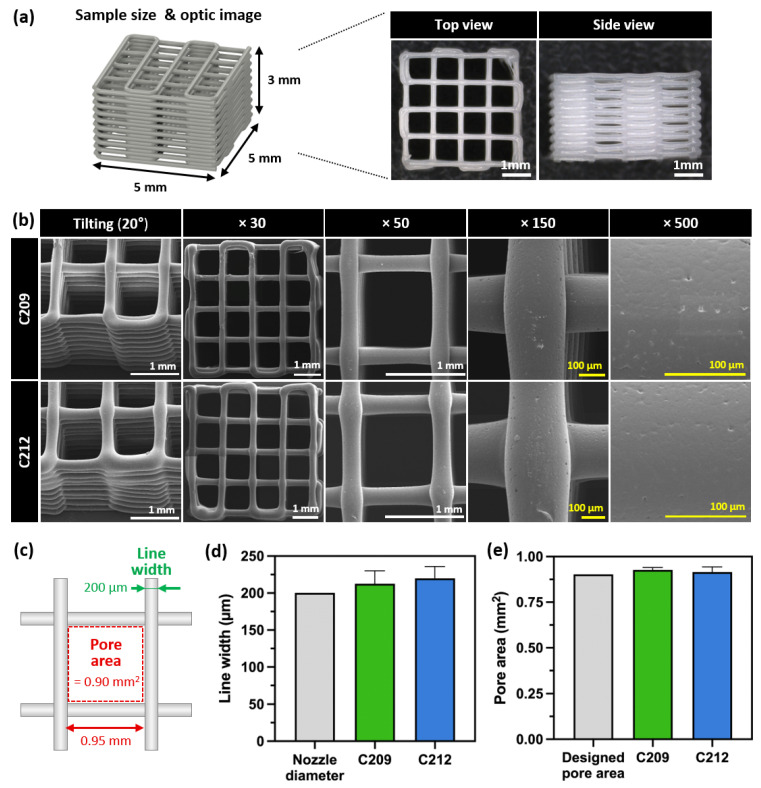
Comparison of printability evaluation: (**a**) Sample dimensions and optic image, (**b**) SEM images of C209 and C212, (**c**) Schematic representation of line width and pore area measurement (designed pore area = 0.90 mm^2^, nozzle diameter = 200 μm), (**d**) Results of line width, and (**e**) Results of pore area.

For the evaluation of printability, lattice scaffolds were fabricated from PCLs as described in Section 2.3.2. C203 exhibited unstable extrusion owing to its low viscosity, whereas C217 failed to extrude molten PCL under pneumatic pressure due to its high viscosity, leading to unsuccessful scaffold fabrication (Figure 3a). Consequently, lattice scaffolds were successfully fabricated using C209 and C212, and their macroscopic and microscopic morphologies were analyzed. Scaffolds fabricated with C209 and C212 maintained structural integrity without collapse or deformation of the designed lattice geometry (Figure 4a). SEM analysis revealed the absence of cracks or voids at both the interlayer junctions and the surfaces, indicating stable stacking into a 3D structure (Figure 4b).

The line width and pore area of the fabricated scaffolds were measured. The scaffolds produced with C209 and C212 exhibited line widths of 212.52 ± 17.58 and 219.84 ± 16.0 μm, respectively, while the pore areas were measured as 0.93 ± 0.01 and 0.92 ± 0.03 mm^2^, respectively (Figure 4c–e). The line widths showed no statistically significant differences compared to the diameter of the precision metal nozzle (200 μm) used, demonstrating the reproducibility of the fabrication process (Figure 4d).

In the pore area analysis, no significant differences were observed among the designed value (0.90 mm^2^), C209, and C212. These findings indicate that the pore structures in both cases were fabricated in close accordance with the designed geometrical specifications (Figure 4e). Therefore, scaffolds fabricated with C209 and C212 were confirmed to achieve precise structural reproducibility despite differences in processing conditions. Quantitative analysis of line width and pore area, together with morphological observations, confirmed the high reproducibility and structural fidelity of scaffolds fabricated from C209 and C212. Overall, these results demonstrate that scaffold integrity can be maintained despite viscosity-driven variations in processing temperature, highlighting the importance of process optimization tailored to material properties.

#### 3.2.3. Evaluation of Mechanical and Chemical Properties After 3D Printing

**Figure 5:**
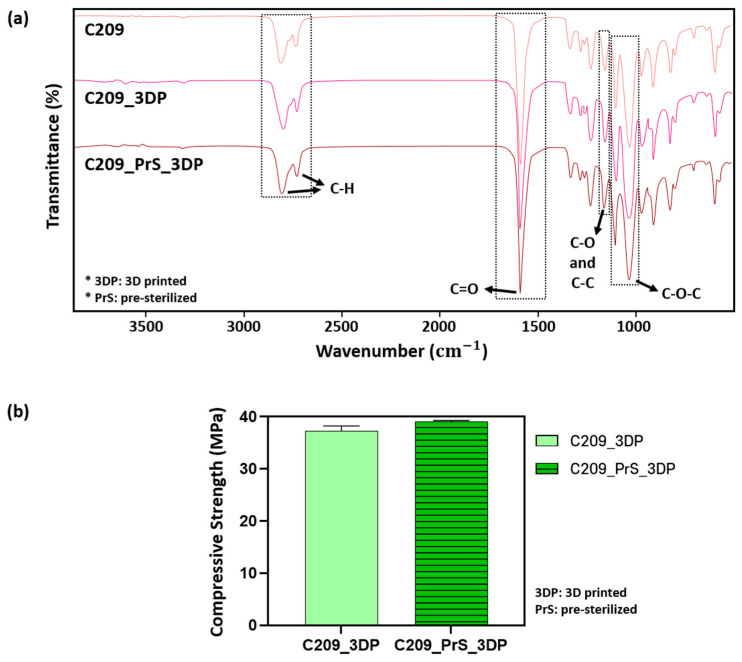
Results of FT-IR spectra and compressive strength after 3D printing: (**a**) FT-IR spectra and (**b**) Compressive strength.

To assess the chemical properties of PCL after 3D printing, C209 scaffolds prepared as described in Section 2.3.2 were selected as representative samples. Comparisons were made among PCL pellets (C209), 3D-printed scaffolds (C209_3DP), and 3D-printed scaffolds fabricated from pre-sterilized material (C209_PrS_3DP). All specimens exhibited characteristic peaks at identical wavenumbers, including ~2940 and ~2865 cm^−1^ (–CH asymmetric and symmetric stretching), 1720 cm^−1^ (C=O stretching), 1290 cm^−1^ (C–O and C–C stretching), and 1160 cm^−1^ (C–O–C stretching), consistent with the intrinsic structure of PCL [37,38,39,40,41,42,43,44]. As shown in Figure 5a, no new peaks or significant shifts were detected, indicating that the 3D printing process did not alter the chemical structure of PCL(Figure 5a).

In addition, to evaluate changes in mechanical properties induced by 3D printing, compressive strength was measured using C209 specimens in the ISO-standard cylindrical form, as described in Section 2.1.2. The compressive strength of 3D-printed scaffolds (C209_3DP) was 37.30 ± 0.95 MPa, while that of scaffolds fabricated from pre-sterilized material (C209_PrS_3DP) was 39.12 ± 0.16 MPa (Figure 5b). A slight increase of approximately 4.9% was observed, which is presumed to result from the evaporation of internal moisture during sterilization. Therefore, maintaining appropriate sealing and humidity control during sterilization may help preserve the mechanical properties of the material even after the sterilization process.

### 3.3. Degradation Behavior and Kinetic Modeling

In this study, pseudo-first-order and pseudo-second-order models were applied to analyze the degradation behavior of medical-grade PCL. The pseudo-first-order model is commonly used in polymer degradation research [45,46,47,48], whereas the pseudo-second-order model has been employed as a complementary approach in some studies [49,50].

The pseudo-first-order model assumes that the degradation rate is proportional to the remaining polymer concentration (or molecular weight), leading to an exponential decrease over time. Typically, this model describes a rapid reduction in the early stage, followed by a gradual deceleration at later stages [51]. As reported by Siparsky et al. and Bhangare et al., polyester polymers such as PCL exhibit hydrolytic degradation that is well described by a pseudo-first-order rate law, and has therefore become the most widely employed kinetic model in this field [52,53]. It was used to interpret the initial degradation behavior of PCL.

The governing equation is expressed as follows:

(2) $$M_{w}(t)=M_{w_{0}}e^{-kt}$$

$M_{w}(t)$ = molecular weight at time *t*

$M_{w_{0}}$ = initial molecular weight

k = degradation rate constant

In contrast, the pseudo-second-order model assumes that the degradation rate is proportional to the square of the polymer concentration, or that it is strongly influenced by factors such as reaction surface area and structural characteristics. Consequently, degradation proceeds gradually at the early stage and accelerates over time. This model is mainly applied in cases where chain scission, changes in molecular weight distribution, or surface accessibility are critical [51,54].

In addition, Middleton and Tipton provided a comprehensive review of the degradation mechanisms of synthetic biodegradable polymers such as PLA, PGA, and PCL. They reported that chain scission and changes in molecular weight distribution substantially affect the degradation rate, in which case the pseudo-second-order model can serve as a complementary approach [55].

The corresponding equation is given as:

(3) $$\frac{1}{M_{w}(t)}=\frac{1}{M_{0}}+k^{'}t$$

$M_{w}(t)$ = molecular weight at time *t*

$M_{w_{0}}$ = initial molecular weight

$k^{'}$ = degradation rate constant

**Figure 6:**
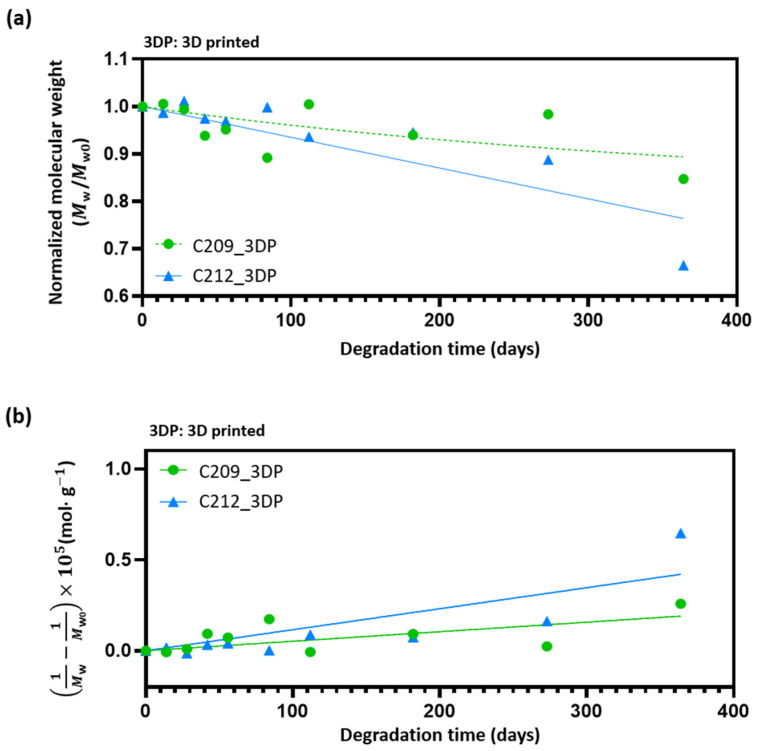
Time-dependent molecular weight ($M_{w}$) reduction in C209 and C212 analyzed using kinetic models: (**a**) Pseudo-first-order and (**b**) Pseudo-second-order.

Among the PCLs investigated, C209 and C212 were selected due to their higher extrusion stability and relatively greater mechanical properties (Figure 3a,b). Scaffolds were fabricated from these grades as described in Section 2.3.2, and their molecular weight changes were monitored over an extended period (0, 14, 28, 42, 56, 84, 112, 182, 273, and 364 days) using GPC analysis (Figure 6a,b). The data were fitted to both pseudo-first-order and pseudo-second-order models, and the kinetic parameters are summarized in Table 3.

Both C209 and C212 showed similar *R*^2^ values in the pseudo-first-order and pseudo-second-order models, with no clear difference in fitting accuracy (Table 3). The purpose of applying these models was not to identify a specific degradation mechanism but rather to verify whether the degradation of medical-grade PCL is influenced by multiple factors rather than a single kinetic pathway [51,54].

### 3.4. Effect of Sterilization on PCL

Scaffolds fabricated by 3D printing must undergo a final sterilization process prior to clinical application [22,56,57]. In this study, E-beam sterilization was employed as it enables rapid processing without residual contaminants. C209 was selected as a representative material, and the effects of sterilization on its chemical and mechanical properties were evaluated.

**Figure 7:**
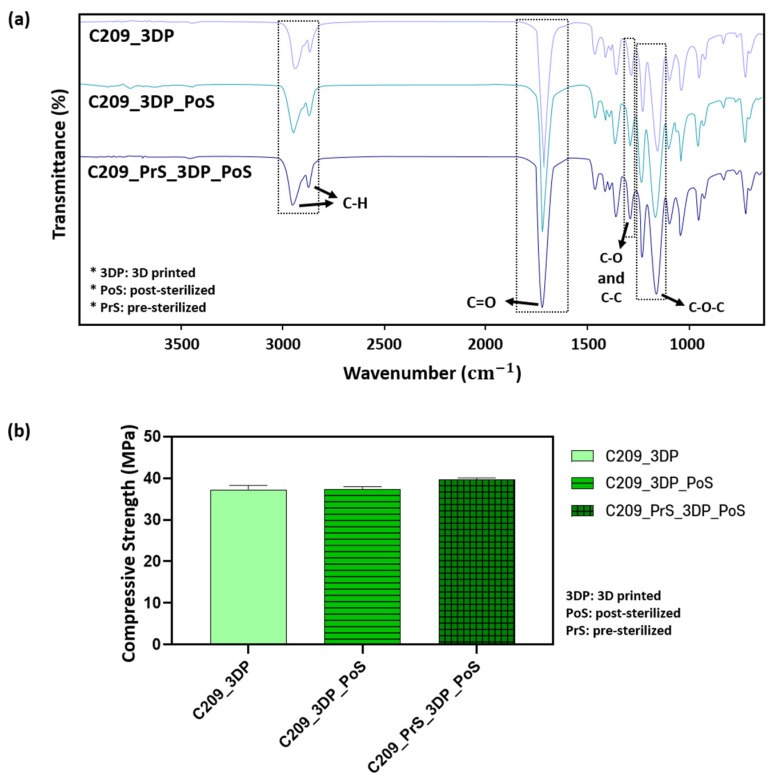
FT-IR analysis and compressive properties of C209 with post-sterilization: (**a**) FT-IR results and (**b**) Compressive strength results.

As shown in Figure 7a. Comparisons were made among 3D-printed scaffolds (C209_3DP), post-sterilized 3D-printed scaffolds (C209_3DP_PoS) and post-sterilized 3D-printed scaffolds fabricated from pre-sterilized material (C209_PrS_3DP_PoS). exhibited characteristic peaks at identical wavenumbers. The major peaks were observed at ~2940 and ~2865 cm^−1^ (–CH_2_ asymmetric and symmetric stretching), 1720 cm^−1^ (C=O stretching), 1290 cm^−1^ (C–O and C–C stretching), and 1160 cm^−1^ (C–O–C stretching), consistent with the intrinsic structure of PCL [37,38]. No new peaks or significant shifts were detected after sterilization, indicating that the process did not alter the chemical structure of PCL (Figure 7a).

The compressive strength of scaffolds sterilized after 3D printing (C209_3DP_PoS) was 37.28 ± 0.70 MPa. For scaffolds fabricated from pre-sterilized material and subjected to an additional sterilization step (C209_PrS_3DP_PoS), the value was 39.70 ± 0.37 MPa.

**Figure 8:**
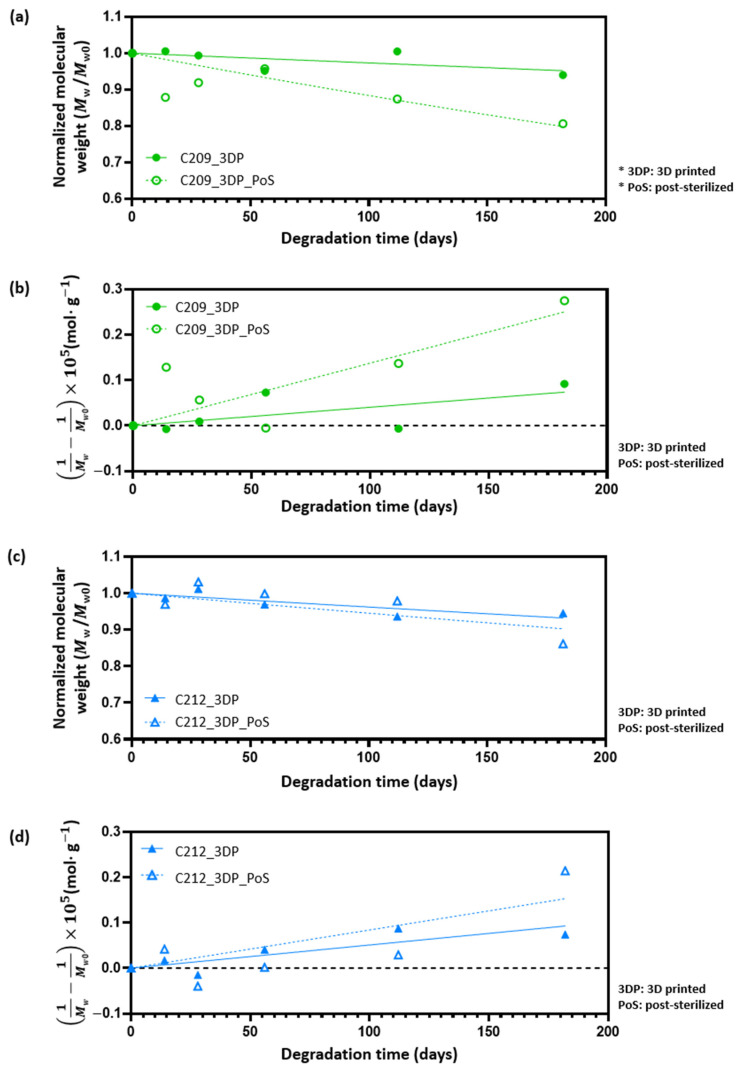
Time-dependent molecular weight ($M_{w}$) reduction in C209 and C212 with and without post- sterilization, analyzed using kinetic models: (**a**,**c**) Pseudo-first-order and (**b**,**d**) Pseudo-second-order.

Although no significant differences were found between these two groups, an increase of approximately 6.4% was observed compared with non-sterilized 3D-printed scaffolds (C209_3DP, 37.3 ± 0.96 MPa) (Figure 7b). However, because the increase was less than 10%, it is unlikely that sterilization induced any substantial changes in the mechanical properties of PCL scaffolds. To further examine whether sterilization influenced the degradation mechanism, molecular weight changes were monitored using GPC over a short-term period (0, 14, 28, 56, 112, and 182 days The data were fitted to pseudo-first-order and pseudo-second-order models (Figure 8), and the kinetic parameters are summarized in Table 4. For both C209 and C212, the coefficients of determination were modest (0.4–0.7), with small differences between the two models (~0.1); pre- and post-sterilization fits were also comparable. This is consistent with the slow bulk hydrolysis of PCL in PBS during the study period, where shallow trends make simple linearized fits sensitive to experimental scatter [51,58]. Prior work under accelerated conditions further shows that the apparent goodness of fit in PCL is strongly model- and condition-dependent [59]. Accordingly, the observed ~0.1 difference in R^2^ lies within expected variability and is not considered meaningful for distinguishing between the two models; likewise, sterilization did not materially affect the overall degradation behavior under our conditions.

### 3.5. Evaluation of Printability Under Long-Term Thermal Exposure

**Figure 9:**
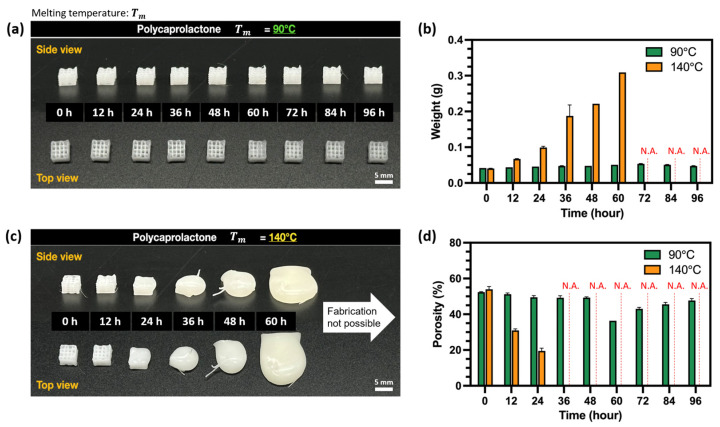
Morphology, weight and porosity of PCL scaffolds under long-term thermal exposure: (**a**) Scaffold morphology at 90 °C, (**b**) Weight variation with time, (**c**) Scaffold morphology at 140 °C, and (**d**) Porosity variation with time.

To investigate the effects of long-term thermal exposure, C209 scaffolds described in Section 2.3.2 were fabricated at 12 h intervals for up to 96 h. At 90 °C, printing was feasible throughout the 96 h period. However, scaffold weight gradually increased with longer melting times, and unstable extrusion was observed after 48 h (Figure 9a,b). This behavior is likely due to partial thermal degradation of PCL during prolonged residence in the barrel, which reduces polymer viscosity and facilitates a higher extrusion flow rate, thereby depositing more material [60]. Consequently, porosity decreased progressively until 48 h, after which it dropped sharply to below 40% due to unstable extrusion (Figure 9c,d).

In contrast, scaffolds printed at 140 °C exhibited a rapid increase in extrusion rate with melting time, and lattice structures could no longer be fabricated after only 12 h (Figure 9c,d). These findings indicate that maintaining consistent scaffold weight, porosity, and morphology requires consideration of thermal degradation time within an appropriate temperature range for each material. Moreover, establishing standardized replacement intervals for the 3D printing material is essential to ensure reproducibility.

## 4. Conclusions

In this study, the mechanical, chemical, and biodegradation properties of medical-grade PCL were systematically evaluated for 3D printing applications. Among the four types of PCL examined, C209 and C212 exhibited superior mechanical strength and stable extrusion behavior, confirming their suitability as materials for 3D printing. SEM analysis and quantitative evaluation further confirmed that both materials were printed uniformly. Biodegradation assessments of C209 and C212 revealed that the 3D printing process and sterilization did not significantly affect their overall degradation behavior. FT-IR and compressive strength analyses of C209, selected as the representative material, also showed that neither the 3D printing process nor E-beam sterilization caused structural or mechanical alterations. Furthermore, printing tests conducted under varying melting times demonstrated that prolonged melting time led to a rapid increase in extrusion rate. This finding suggests that, to maintain consistent scaffold morphology and porosity, the thermal degradation time within the appropriate temperature range for each material must be carefully considered. Taken together, these results demonstrate that C209 and C212 remain stable even after sterilization and are promising candidates for 3D printing applications. This study provides fundamental evidence supporting the clinical translation of medical-grade PCL scaffolds for tissue engineering and biomedical implantation.

## Figures and Tables

**Table 1:** Type of medical-grade PCLs.

Polymer Name	Inherent Viscosity (dL/g)	Composition	End Group	Company (Country)
RESOMER^®^ C203	0.35–0.43	Poly (ɛ-caprolactone)	Ester	Evonik (Germany)
RESOMER^®^ C209	0.8–1.0			
RESOMER^®^ C212	1.13–1.38			
RESOMER^®^ C217	1.53–1.87			

**Table 2:** Classification of specimens by sterilization condition.

Specimen Name	Pre-Sterilization	Post-Sterilization
PCL-3DP ^1^	No	No
PCL-PrS ^2^ -3DP	Yes	No
PCL-3DP-PoS ^3^	No	Yes
PCL-PrS-3DP-PoS	Yes	Yes

^1^ 3DP: 3D-printed, ^2^ PrS: pre-sterilized, ^3^ PoS: post-sterilized. Note: “PCL” in specimen names refers to medical-grade PCLs (C203, C209, C212, and C217). The classification described here was consistently applied across all subsequent analyses, including mechanical testing, FT-IR, and degradation experiments.

**Table 3:** Kinetic parameters (*k*, *k’*) and coefficients of determination ($R^{2}$) of C209 and C212 obtained from pseudo-first-order and pseudo-second-order models.

Kinetic Model	Material	k (day^−1^)	$R^{2}$
Pseudo-First-Order	C209	$2.249\times 10^{-3}$	0.298
	C212	8.294 × 10^−7^	0.770
**Kinetic Model**	**Material**	$k^{'}$ **(day^−1^)**	$R^{2}$
Pseudo-Second-Order	C209	5.236 × 10^−4^	0.326
	C212	1.156 × 10^−3^	0.695

**Table 4:** Kinetic parameters (k, $k^{'}$) and coefficients of determination ($R^{2}$) of C209 and C212 with and without post-sterilization on obtained from pseudo-first-order and pseudo-second-order models.

Kinetic Model	Material	Post-Sterilization	k (day^−1^)	$R^{2}$
Pseudo-First-Order	C209	No	$1.2\times 10^{-3}$	0.399
		Yes	$0.2\times 10^{-3}$	0.413
	C212	No	$0.4\times 10^{-3}$	0.715
		Yes	$0.6\times 10^{-3}$	0.604
**Kinetic Model**	**Material**	**Post-Sterilization**	$k^{'}$ **(day^−1^)**	$R^{2}$
Pseudo-Second-Order	C209	No	$0.4\times 10^{-3}$	0.408
		Yes	$1.3\times 10^{-3}$	0.642
	C212	No	$0.5\times 10^{-3}$	0.715
		Yes	$0.8\times 10^{-3}$	0.625

## Data Availability

The data are contained within the article.
